# Gain-of-Function (GOF) Mutant p53 as Actionable Therapeutic Target

**DOI:** 10.3390/cancers10060188

**Published:** 2018-06-07

**Authors:** Ramona Schulz-Heddergott, Ute M. Moll

**Affiliations:** 1 Institute of Molecular Oncology, University of Göttingen, 37077 Göttingen, Germany; utemarthamoll@gmail.com; 2 Department of Pathology, Stony Brook University, Stony Brook, NY 11794, USA

**Keywords:** mutant p53 (mutp53), missense p53, gain-of-function (GOF), p53 loss-of-heterozygosity (LOH), drug therapy, HSP90, HSF1

## Abstract

p53 missense mutant alleles are present in nearly 40% of all human tumors. Such mutated alleles generate aberrant proteins that not only lose their tumor-suppressive functions but also frequently act as driver oncogenes, which promote malignant progression, invasion, metastasis, and chemoresistance, leading to reduced survival in patients and mice. Notably, these oncogenic gain-of-function (GOF) missense mutant p53 proteins (mutp53) are constitutively and tumor-specific stabilised. This stabilisation is one key pre-requisite for their GOF and is largely due to mutp53 protection from the E3 ubiquitin ligases Mdm2 and CHIP by the HSP90/HDAC6 chaperone machinery. Recent mouse models provide convincing evidence that tumors with highly stabilized GOF mutp53 proteins depend on them for growth, maintenance, and metastasis, thus creating exploitable tumor-specific vulnerabilities that markedly increase lifespan if intercepted. This identifies mutp53 as a promising cancer-specific drug target. This review discusses direct mutp53 protein-targeting drug strategies that are currently being developed at various preclinical levels.

## 1. Introduction

The tumor suppressor TP53 is the single most frequently mutated gene in over 50% of all human cancer patients. Alterations in p53 signaling pathways are required for development of most cancers. Unlike other tumor-suppressors, missense mutations within the DNA-binding domain represent 75% of all p53 alterations, including six most frequent ‘hotspot’ codons that encompass ~30% of all missense mutations [1], leading to highly stabilized mutant p53 proteins (mutp53). Missense mutations either reduce, alter, or preclude interaction of p53 with its consensus DNA-binding sequence, and thus cannot activate the p53 tumor suppressive transcription response.

Importantly, mouse models and clinical data from germline and sporadic cancers have now firmly established that p53 missense mutations not only abolish the tumor suppressive function, but also often acquire new tumorigenic driver activities (gain-of-function, GOF) [2,3,4,5]. Functionally, neomorphic mutp53 promotes multiple oncogenic pathways, impacting transcriptional regulation, chromatin structure, replication of DNA microRNA biogenesis, and altering the proteome and metabolic pathways [6,7,8,9,10]. Mutp53 GOF proteins accumulate to high levels in the nucleus. A major mechanism how mutp53 profoundly affects broad genomic and transcriptomic changes to mediate GOF strongly points to mutp53 working by physical recruitment to other transcription factors and to the chromatin remodeling complex SWI/SNF, thereby profoundly altering numerous cellular programs that accelerate tumor progression and metastasis. The expanding network of mutp53 protein interactions profoundly remodels the cancer cell transcriptome and proteome. Thus, p53 GOF mutants subvert a remarkable plethora of molecular pathways to reprogram cell behavior and promote cancer cell survival and proliferation, invasion and migration, stem cell renewal, chemoresistance, stroma remodeling, and inflammation. A central point is that all these new acquired functions strengthen the addiction of cancer cells to the continued presence of high levels of mutp53. This addiction can be therapeutically exploited by strategies to eliminate or disable it. Functional inhibition of mutp53 was shown to effectively prevent cancer growth in vitro and in xenografts [11,12,13,14]. We recently showed that acute genetic ablation or pharmacologic degradation of mutp53 in autochthonous tumors triggers strong cytotoxicity in different hotspot GOF knockin mice, translating to suppression of tumorigenesis and major gains in animal survival by up to 59% [15]. This antineoplastic effect of mutp53 removal operates in the absence of wild-type p53 (wtp53). This is human-relevant, since the majority of human mutp53 tumors undergo loss-of-heterozygosity (LOH). GOF mutants represent a phenotypic spectrum and depend on tumor context, described in several excellent recent reviews [6,7,16,17,18,19,20]. Taken together, targeting cancer-associated mutp53 GOF functions is a highly promising rational approach that strikes cancer cells selectively, with low toxicity in healthy tissues. Given the remarkably high frequency of TP53 missense mutations across all cancer types, this therapeutic concept appears to be broadly applicable for patients worldwide.

Another function of mutp53, widely observed in ectopic mutp53 overexpression cell culture models, is the so-called dominant-negative (DN) effect of mutp53 over all family members, i.e., wild-type p53, TAp63 and TAp73, to inhibit their tumor suppressive functions. However, consistently in vivo in Li-Fraumeni patients and mouse models, a DN effect is either not observed or not pronounced and highly context dependent. In a study characterizing 104 Li-Fraumeni patients, no evidence of DN was seen. Instead, the degree of transactivation deficiency (based on a functional yeast assay) and haplo-insufficiency of the p53 alleles are the driving factors for cancer proneness in patients [21,22]. In further support of this notion, in some spontaneous and KrasG12D-induced heterozygous mouse tumor models, the remaining endogenous wild-type p53 allele shows a dominant-positive tumor suppressor effect over the missense mutp53 allele and activates broad transcriptional wild-type p53 activities and response [23,24]. This dominant-positive/tumor-suppressive effect of wild-type over mutant p53 might be the force behind the high occurrence of p53 loss-of-heterozygosity (LOH) in mutant tumors [19,24]. It further suggests that ectopic in vitro models might be at least in some cases an artifact of severe overexpression not mimicking the endogenous condition.

### 1.1. Missense Mutant p53 Stabilization

The stabilization of mutp53 proteins is a prerequisite for the manifestation of the gamut of their gain-of-function (GOF) properties [25,26,27]. Of note, stabilization is not an intrinsic characteristic of missense p53 mutants, but rather an external tumor-cell specific event.

Mutp53 stabilization can be readily explained for the large class of unstable conformational mutants (also called structural mutants, for example at residues R175, G245, R249 and R282). Conformational mutations lower the melting temperature and cause protein destabilization, which in turn leads to abnormal folding [1,18]. Stabilization is somewhat harder to explain for contact mutants (for example R248 and R273) because they are thought to largely maintain the wild-type conformation in the scaffold backbone of their DNA binding domain, while surface residues important for DNA binding are altered. Importantly, the main route towards stabilizing both classes of mutp53 goes over MDM2, the main E3 ubiquitin ligase for p53, specifically via the escape from MDM2-dependent degradation [6,28].

### 1.2. Aberrant Stabilization of mutp53, a Cancer-Specific Trait Absent in Normal Cells, Is Caused by Inactivation of MDM2 and CHIP

The old dogma on how mutp53 escapes MDM2-mediated degradation had reasoned that mutp53 accumulates to high levels because of the loss of wild-type p53-dependent transcription of MDM2. The observed increase in cellular half-life underlies the description of mutp53 as a ‘stable’ protein. Importantly, Mdm2 is also the principal mutp53 E3 ligase in vivo [27]. Mutp53 in normal tissues of KI mice is inherently unstable due to efficient degradation by baseline levels of Mdm2, generated p53-independently from Mdm2’s constitutive P1 promoter. Thus, KI mice firmly established that their normal tissues have adequate enzymatic activities of Mdm2 and other E3 ligases to sustain control over mutp53 to match the low levels of wtp53. This eliminated the long-held notion that mutp53’s transcriptional inability to activate Mdm2 as p53 target gene via the Mdm2 P2 promoter is the cause for mutp53 hyperstability. Instead, mutp53 proteins undergo marked constitutive stabilization exclusively in tumors [2,27]. Thus, upon malignant conversion, a second alteration must occur that inhibits baseline Mdm2 and other E3 ligases, thereby stabilizing mutp53.

Hints to answer the mystery of mutp53 stabilization came from the old observation that wild-type p53 undergoes transient interactions with elements of the heat-shock protein (HSP) chaperone machinery. Importantly, these studies also identified stable interactions between mutp53 and the constitutively expressed Hsc70, or stress-induced Hsp70 and Hsp90 components of HSP [29,30,31]. Over the years, the question of how mutp53 escapes MDM2-mediated degradation in tumors also became clearer by involving the chaperone machinery [31,32,33,34]. The chaperone-associated E3 ubiquitin ligase CHIP (C terminus of Hsc70-interacting protein) is a key component of chaperone complexes, and normally marks aberrant and misfolded polypeptides for degradation. It was first shown that CHIP induces proteasomal degradation of both wild-type p53 and mutp53. CHIP-induced degradation of mutp53 was associated with the Hsc70 and Hsp90 chaperones [35]. Lukashchuk et al. 2007 then extended this observation by showing that mutant and wild-type p53 proteins were ubiquitinated and degraded through overlapping but distinct pathways [36]. Here, CHIP was only able to ubiquinate mutp53, while Mdm2 has driven the degradation of both mutant and wild-type p53 [36]. We established that endogenous mutp53 in human cancers suffers from a profound lack of ubiquitination—the root cause of its degradation defect—despite detectable (albeit lower) Mdm2 levels and normal Mdm2 interactions. By up/down adjustments of Mdm2 levels, we showed that Mdm2 E3 ligase activity is specifically inhibited in mutp53 cancer cells [37].

Guarding the proteome against misfolding and aggregation, molecular chaperones from the HSP machinery are essential to guide proper folding of polypeptides into mature proteins. The multi-component HSP chaperone machinery is a powerful stress-induced system required to support the cancer state [38,39,40,41]. The reason is that cancer cells are in a permanent state of proteotoxic stress due to cell-extrinsic (e.g., hypoxia, acidosis) and cell-intrinsic (e.g., aberrant/misfolded oncoproteins, massive oxidative stress, high levels of DNA damage) aberrant conditions. Tumor cell proteins, especially their mutated and deregulated oncoproteins, require constant massive chaperone support, especially from the HSP90 system, to prevent illicit interactions and toxic protein aggregation [39,42,43]. Other important HSP chaperones that counteract proteotoxic stress are the HSP70 complex, which involves the HSP40/DNAJ system [44]. Specifically, the HSP70/HSP40 system takes on a legwork-performing preparatory role, transferring polypeptides to other systems such as the HSP90 system. In cancer cells, super-chaperone complexes, including HSP90/HSP70 and members of the HSP40/DNAJ family, are the major forms, protecting aberrant proteins from degradation [45,46,47].

Importantly, the normal HSP90 function is subverted during oncogenesis to enable maintenance of malignant transformation (‘co-oncogenesis’). Cancer cells become addicted to the HSP machinery, turning it into a powerful pro-survival and anti-apoptotic system. Consequently, HSP90 is abberantly upregulated and hyperactivated, especially in cancers but not in normal cells, generating a cancer-specific therapeutic window [41,48]. Mechanistically, tumor cells respond to many kinds of stresses with the activation of heat-shock factor 1 (HSF1), the master transcription factor for all stress-induced heat-shock proteins which include Hsp90α, Hsp70, members of the HSP40/DNAJ family, and numerous co-chaperones [49,50]. HSF1 is a key determinant during oncogenesis shown in several mouse cancer models. HSF1 knockout mice are remarkably resistant to tumor induction by oncogenes [38,51,52] through e.g., modulating the expression of a broad set of genes involved in cell-cycle regulation, signaling, metabolism, adhesion, and protein translation. The HSF1-HSP axis plays a key role in stabilization of tumor-promoting proteins and oncogenes, including receptor tyrosine kinases (ErbB1/EGFR and ErbB2/HER2) [53,54], signaling kinases (Bcr-Abl and AKT) [55], pro-inflammatory cytokines [56], and mutp53 [33,37].

Thus, in contrast to wtp53, most mutp53 proteins are conformationally aberrant and depend on stable complexes with the multi-component chaperone machinery to avoid aggregation. We showed that stable mutp53-HSP90 interaction strongly protects mutp53 from endogenous Mdm2 and CHIP E3 ligase activity [37]. Both structural as well as DNA-contact p53 mutants are dependent on HSP90, preventing their degradation by Mdm2 and CHIP. Interference with the HSP90 system by depleting the Hsp90 core protein, or by pharmacological inhibition of the Hsp90 ATPase activity with the competitive ATP pocket inhibitor 17AAG abolished the complex, released mutp53 and reactivated endogenous Mdm2 and CHIP. This degrades mutp53 but not wtp53. Conversely, 17AAG-mediated mutp53 destabilization was rescued by Nutlin or knockdown of Mdm2 and CHIP. Thus, 17AAG efficiently degraded mutp53 protein, which caused a strong loss of cell viability in mutp53 cancer cells, but not in wtp53 cancer cells. In support of a causal link, the killing efficacy of 17AAG was overwhelmed by additional overexpression of mutp53 [37]. Thus, a major determinant of mutp53 stabilization is a functional HSP90 machinery.

Interestingly, mutp53 forms a positive feed-forward loop to Hsp90, which further reinforces mutp53 stability. In HER2+ breast cancer, GOF mutp53-R175H triggered HSF1 activity, which leads to further upregulation of the heat shock response via Hsp90/Hsp70, which in turn further stabilizes Hsp90 clients including mutp53 and HER2 [57]. Moreover, overexpressed HER2 receptor regulates the HSF1 response via the PI3K/AKT/mTOR pathway [58]. This loop contributes to driving HER2+ breast cancer. Importantly, Hsp90 inhibitors are currently in clinical evaluation for cancer and other diseases.

By further investigating the pathways that protect mutp53 from ubiquitin-mediated degradation, Ingallina et al. showed that Hsp90/HDAC6-regulated mutp53 stabilization is sustained by an axis encompassing the mevalonate pathway promoting RhoA geranylgeranylation, membrane insertion and transduction of mechanical signals from the extracellular environment [59]. Mutp53 levels can be heterogeneous even within the same tumor [27]. The authors speculate that the spatial heterogeneity of mutp53 accumulation observed in some tumors might be due to differential mechanical niches within the tumor tissue.

Parrales et al. [60] identified the Hsp40 isoform DNAJA1 as another chaperone promoting stabilization of misfolded conformational mutp53 proteins by physical interaction, protecting mutp53 from CHIP-mediated degradation. Protection of mutp53 by DNAJA1 was dependent on the mevalonate pathway metabolite MVP. Reduced MVP levels—through manipulating the mevalonate pathway genetically or via Statins—is thought to change mutp53’s binding partner from DNAJA1 to CHIP by changing mutp53 conformation, although the mechanism is not well understood [60]. Of note, HSP40/DNAJ family members regulate the HSP70 chaperone system through stimulation of the basal Hsp70 ATPase activity and substrate affinity. Moreover, the core components Hsp70/Hsp40 and Hsp90 are found in the same cancer-associated super-chaperone complexes and might stabilize mutp53 in a single complex.

A completely new angle of mutp53 stabilization might come from MDM2 splice isoforms. MDM2-B, the most frequently overexpressed splice isoform in human tumors, binds to full-length MDM2 to interfere with MDM2-induced mutp53 degradation. This tumor-promoting MDM2-B function leads to mutp53 accumulation and GOF activity in murine cancer models. Moreover, overexpressed MDM2-B correlated with mutp53 stability in human tumors. Such dominant-negative MDM2 isoforms might contribute to HSP90-mediated inactivation of full length MDM2 [61].

We identified a second major stabilization determinant in tumors that are initially p53 heterozygous (mut/wt). We showed that the wtp53 allele exerts a repressive function regarding mutp53 stabilization in vivo. In mouse tumors with high frequency of p53 LOH, mutp53 protein was stabilized (94% of cases) and GOF manifested. Conversely, in mouse tumors with low frequency of p53 LOH, mutp53 was not stabilized (80% of cases) and GOF not observed. Human genomic databases show a high degree of p53 LOH in all examined tumor types that carry p53 missense mutations. Thus, in heterozygosity LOH is a critical prerequisite for mutp53 stabilization and GOF in vivo [25].

In sum, these studies clearly show that mutp53 stabilization is a tumor-specific vulnerability, which is pharmacologically tractable. Therefore, continued efforts to comprehensively understand the mechanisms of mutp53 stabilization remain crucial towards translation into therapy.

## 2. Targeting mutp53 for Cancer Treatment

We are currently uncovering an ever expanding network of mutp53 protein-protein interactions that subvert a remarkable number of normal regulatory pathways to promote cancer cell proliferation and survival, aggressiveness, invasion, metastasis, chemoresistance, and tissue remodeling. Thus, finding routes to eliminate mutp53, or reactivating mutp53 to regain wild-type activity is highly relevant for therapy. Re-expression of the wild-type p53 allele is sufficient to induce spontaneous tumor regression in p53null mice, while it is at least halting tumor progression in mutp53-R172H/R172H mice due to a dampening effect of the mutp53-R172H towards the wild-type protein [22,62]. Even more encouragingly, in KrasG12D/p53 mouse models of lung adenocarcinoma, switching back the remaining wild-type p53 allele at the endogenous locus induces a strong tumor-suppressive response in p53-null as well as in mutp53-R172H and -R270H lung tumor cells in 3D cultures and in vivo [24].

**Figure 1:**
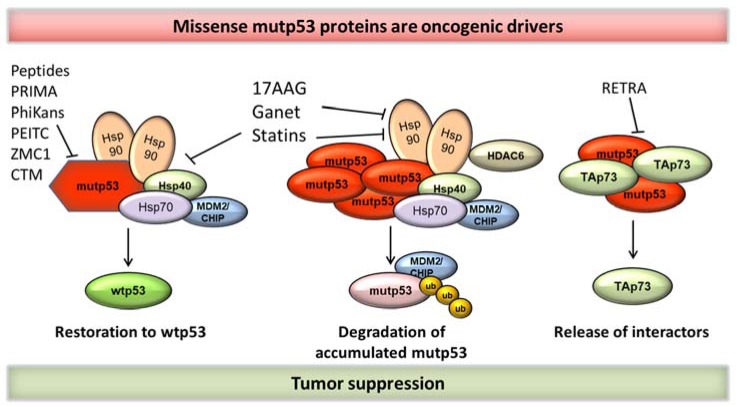
Strategies to target missense mutant p53 proteins. These approaches are currently being explored to target mutated p53 (mutp53). (**left**) Small molecules or small peptides might restore p53 to its wild-type-like conformation and regain tumor suppressive functions. Some of these compounds also partly degrade stabilized mupt53 levels; (**middle**) induction of mutp53 degradation by inhibition of the Heat-shock protein (HSP) chaperone machinery. Compounds can target different parts of the super-chaperone complexes, including Hsp90-Hsp40 and histone deacetylase 6 (HDAC6). Targeting these complexes leads to reactivation of E3 ubiquitin ligases such as MDM2 and/or CHIP to induce missense p53 protein degradation (loss of mutp53 protection); (**right**) Another approach involves the hetero-complexes between p53 family members. mutp53 can inhibit tumor suppressive members such as TAp73. Small molecules destroy such complexes to release TA73.

Thus, for the two last decades, numerous compounds were identified to destabilize highly accumulated GOF p53 mutants, or to reverse the oncogenic properties of mutp53 (Figure 1 and Table 1) (also reviewed in [6,7,19,20,63]). Besides strategies to directly target stabilized mutp53 for degradation or reactivation by restoring a ‘wild-type-like’ conformation, many approaches are indirect, suggesting to (I) exploit tumor cell vulnerabilities that result from missense mutp53-specific signaling to downstream pathways [7,20,64], (II) inhibit the remaining G2 checkpoint on which such tumor cells depend, since wtp53-deficient tumors have lost their G1 checkpoint [20,64], (III) target mutp53-mediated metabolic pathways [64,65] and (IV) inhibit mutp53 interactors that accelerate cancer progression [7,20,64].

### 2.1. Strategies to Target mutp53—Induced Degradation

GOF mutant p53 proteins, in particular when highly stabilized, are prone to strategies that induce mutp53 degradation. All such strategies suggested so far involve the chaperone machinery.

#### 2.1.1. Inhibition of the HSP90/HDAC6 Axis

As discussed above, a major determinant of mutp53 stabilization is a functioning HSP90 machinery [37,63,80]. Importantly, inhibition of Hsp90 alone, with 17DMAG or Ganetespib, or in combination with its obligatory regulator cytosolic HDAC6, has marked anti-tumoral effects in vivo and extends the overall survival of mutp53-R175H and mutp53-R248Q knockin mice by 30–59%. Remarkably, although the mentioned drugs are pleiotropic drugs, p53-null control mice did not benefit from Hsp90 inhibition. These anticancer effects are concomitant with mutp53 degradation and cancer cell death, indicating tumor addiction to highly stabilized mutp53 [15].

#### 2.1.2. Hsp40 Inhibitor Chetomin

Chetomin, a fungal-derived compound identified in a cell-based screen, is capable of specifically reactivating p53-R175H to wild-type-like activity [79]. The anticancer activity of Chetomin was evaluated in human cancer cell lines with mutated p53 such as p53-R175H and p53-R273H, wtp53, p53null and in normal cells. Chetomin was most cytotoxic—albeit to various extent—to various p53-R175H cell lines, inducing p53 target genes such as MDM2, p21 and Bbc3/Puma. Minimal or no induction was seen in cancer cells with other missense p53 mutations, wtp53 or p53null. Mouse xenografts confirmed the allele-specific higher response of p53-R175H tumors to Chetomin by decreased tumor volume and weight. Chetomin failed to inhibit p53-R273H or p53null tumors. Mechanistically, Chetomin binds to Hsp40 (but not to Hsp90) and increases its capacity to p53-R175H protein, presumably then causing a conformational change to a wt-like folding.

#### 2.1.3. Statins

Freed-Pastor and co-workers first identified the mevalonate/sterol biosynthesis pathway to be upregulated by mutp53, and responsible for the disordered and invasive morphology of mutp53 breast cancer mammospheres [11]. Mutp53 knockdown greatly improved this malignant phenotype. Mechanistically, mutp53 bound to sterol gene promoters via the SREBP1/2 transcription factors. This implicated the mevalonate pathway as a therapeutic target in mutp53-haboring cancers, generating the idea to inhibit the pathway by statins. Indeed, statins phenocopied the improvement seen by mutp53 knockdown, suppressing invasion and proliferation and inducing extensive cell death.

High-throughput screening of the FDA-approved drug library independently identified statins as degradation inducers of misfolded mutp53 proteins of the conformational class (shown for p53-R156P, p53-Y157F, p53-R175H and p53-Y220C) with minimal effects on wtp53 levels. Statins preferentially inhibited the growth of mutp53-expressing tumor cells in vitro and in xenografts. Reduction of mevalonate-5-phosphate (MVP) by statins or knockdown of the mevalonate kinase induced CHIP-mediated mutp53 degradation by disruption of mutp53/DNAJA1 protein complexes. DNAJA1, an Hsp40 isoform, also protects mutp53, as previously shown for Hsp90. However, the precise mechanism how statin liberates mutp53 from the protective interaction with DNAJA1 remains elusive. Thus, depletion of mutp53 by mevalonate pathway inhibition holds promise for statins in cancer therapy [60]. Which specific missense mutp53 proteins might be susceptible and in what context, however, remains to be defined, based on current results. In an orthotopic KrasG12D-driven mouse lung tumor model, a therapeutic response to simvastatin was selectively identified in p53-R270H contact mutant tumors, but not in conformational p53-R172H mutant tumors [24].

Ingallina et al. also independently identified statins as drugs that exclusively degrade mutp53 proteins by disrupting the SREBP-mevalonate / RhoA mechano signalling pathway, which controls Hsp90-mediated mutp53 stabilization [59]. Here, statin leads to the dissociation of Hsp90 from mutp53 with subsequent reactivation of MDM2, suggesting that mevalonate pathway inhibition in tumors reinstates the instability of mutp53. This statin action appears to be linked to its ability to impede HDAC activities in cancer cells, including the Hsp90-associated HDAC6 [81].

Although many clinical studies support a tumor-suppressive role of statins in human cancer, others are inconclusive or fail to show a benefit [82,83]. Preclinical data demonstrate encouraging anticancer activities of statins. Interestingly, retrospective studies show chemoprevention and survival benefit by statins in several types of cancer. However, the survival benefit could not be confirmed in prospective clinical trials due to the lack of well-conducted large-scale phase III randomized controlled trials that address the antitumor effects of statins. Moreover, the TP53 tumor status was not considered, although it might be an important predictor of the response to mevalonate pathway inhibitors (reviewed by [19]). Re-analysis of these studies using TP53 mutational stratification might give a clearer picture. 

The work of Ingallina et al. [59] (involving Hsp90 and MDM2), the Moll group [37] (involving Hsp90, MDM2 and CHIP), Hiraki et al. [79] (involving Hsp40 and MDM2) and Parrales et al. [60] (involving Hsp40 and CHIP) defines related pathways to stabilize mutant p53 by the HSP chaperone system, suggesting possible synergism. It would therefore be interesting to test Hsp90 inhibitors and statins or Hsp90 and Hsp40 inhibitors in combination.

### 2.2. Strategies to Target mutp53—Reactivation of Wild-type-Like p53 Activity

Restoration of wild-type p53 functions such as apoptosis and senescence are a priori an attractive idea to revert mutp53 into wtp53 properties, especially since mutp53 proteins generally occur at higher levels and thus might serve as reservoir for reactivatable p53. However, such ‘reactivators’ are mostly at the level of preclinical testing.

#### 2.2.1. PRIMA-1 and PRIMA-1^Met^ (APR-246)

One of the oldest and most intensively studied reactivator compounds, now in clinical trials, is PRIMA-1 (‘p53 reactivation and induction of massive apoptosis’; chemical name: 2,2-bis (hydroxymethyl)-1-azabicyclo[2.2.2]octan-3-one) [66] and its derivate PRIMA-1Met (APR-246) [67]. PRIMA-1 was identified in a cell-based screen of the NCI drug library for its ability to restore select p53 missense mutants to wtp53 properties, assessed by inducing p21, cell cycle arrest and apoptosis [66]. The methylated form of PRIMA-1, PRIMA-1^Met^ (APR-246), is more effective and less toxic [69]. Chemically, PRIMA-1 is a prodrug and converted in tumor cells to the active metabolite methylene quinuclidinone (MQ), which binds to cysteine residues of mutp53 proteins [68]. Binding of MQ is thought to cause proper mutp53 refolding to wtp53. Evidence for refolding to wtp53 is indirect through e.g., differential interaction with p53 conformation-specific antibodies. PRIMA-1 and APR-246 re-established wtp53-like transcriptional activity with increased expression of Puma, Noxa and Bax target genes [68,84] and impressive cytotoxic and apoptotic responses in mutp53 cells in vitro [85,86]. PRIMA-1 was found to restore wtp53 properties on most of the tested mutp53 proteins [66]. However, it was also reported that PRIMA-1 and APR-246 have p53-independent actions. A detailed review on all PRIMA-1 and APR-246 actions is given by Krayem and coworkers [87]. Clinical trials with APR-246 are ongoing, e.g., a Phase Ib/II study in platinum-sensitive recurrent high-grade serous ovarian cancer combined with carboplatin and Doxorubicin (NCT02098343), and a Phase II study in platinum-resistant high-grade ovarian cancer combined with Doxorubicin (NCT03268382). Preliminary safety assessment suggests that APR-246 is relatively non-toxic. However, questions remain. This includes e.g., the specificity for MQ to bind to different mutp53 forms and the p53-independent drug activity. Still, this relatively non-toxic drug, potentially able to target all GOF mutp53 proteins in solid cancers, remains a promising route.

#### 2.2.2. Small-Molecule Stabilizers of the p53-Y220C Mutant

The Y220C mutation in the p53 core domain creates a solvent-accessible cleft that destabilizes the protein and leads to loss of DNA binding [88]. Fersht et al. identified PhiKan083 and PhiKan7088 to bind specifically to this cleft and restore wild-type p53 folding in cultured cells [70]. In vitro, these stabilizers corrected p53-Y220C shown by cell cycle arrest and apoptosis via restoration of p21 and Noxa expression, respectively [71]. PK7088 works synergistically with Nutlin-3a to further increase p21 and Noxa expression, which confirms the p53-Y220C conformational rescue. However, the anti-cancer activity of PhiKan7088 remains to be shown in animal models.

#### 2.2.3. mutp53 Reactivation by the Dietary Compound PEITC

PEITC (cruciferous-vegetable-derived phenethyl isothiocyanate) is a natural compound that is known to inhibit proliferation and induce apoptosis in cancer cells. Previous studies showed PEITC-induced apoptosis without consideration of the p53 status. Later, PEITC was also shown to impair cancer growth independent of p53 status [89]. Treatment with PEITC induced ROS (reactive oxygen species), GSH depletion and a DNA damage response via p53 activation and p53 target gene expression [89]. PEITC reactivated p53 mutants in vitro and in vivo and preferentially demonstrated growth-inhibitory activity in p53-R175H vs. p53-R273H and p53-R248Q tumor cells [72]. PEITC induced apoptosis by enhancing p53 target gene expression of e.g., p21, MDM2, Puma, Noxa, Bcl-2 and Bax. SKBR3 breast cancer xenografts (expressing p53-R175H) fed with a PEITC-containing diet showed inhibited tumor growth associated with 21 and Bax induction. The mechanism underlying PEITC actions and its signaling pathways are not completely understood. However, mutp53 reactivation by a dietary compound might be an actionable route for cancer prevention.

#### 2.2.4. Small Peptides to Target mutp53 Aggregation

The hypothesis underpinning the strategy of small peptides is the notion that mutp53 proteins exist in a transient and dynamic equilibrium between misfolded and properly folded conformations. Small peptides bind preferentially to mutp53 during their transient wild-type state, thereby stabilizing its native folding and shifting the population equilibrium to the wild-type p53 state [1]. This may explain the fact that several small peptides bear similarities to known p53 interacting proteins. Peptides were generated with different molecular sizes and sequences, binding to different p53 regions. Thus, small peptides differ in their mechanism of action to reactivate mutp53 [90].

ReACp53 was reported to prevent the amyloid-like aggregation of mutp53 proteins harboring either mutations in p53-R248Q or p53-R175H. Reactivated p53 acts in the same manner than its wild-type counterpart and regulated p53 target gene expression, reduced cell proliferation and increased cell death. Treatment of ovarian carcinoma-bearing mice with conjugated ReACp53 caused decreased proliferation and tumor reduction [73]. Interestingly, ReACp53 treatment resulted in lower p53 levels due to subsequent degradation of the wtlike folded protein by MDM2.

Tal et al. selected mutp53-R175H and mutp53-R249S reactivating small peptides that were randomly generated by phage display libraries [74]. Lead peptides (pCAPs) were produced and evaluated for their ability to restore proper p53 folding and activity. Several pCAPs restored the ability of p53 to bind to its response elements DNA sequence-specific binding and to induce p53 target gene expression concomitant with apoptosis selectively in mutp53-habouring cancer cells. Mixtures of different pCAPs, used to inject different mouse xenograft cancer models expressing mutp53, showed tumor regression in cancer types such as colorectal, ovarian, and breast cancer [74].

One advantage of peptides is their ability to specifically bind their targets. Another advantage is the low risk of small peptides exclusion from cells by multiple drug resistance mechanisms. On the other hand, the general disadvantage of peptides as therapeutics is their chemical and physical instability. In addition, depending on their amino acid composition, they may exhibit low membrane permeability, a problem which can be partly prevented by using chemical modifications [91].

#### 2.2.5. Zinc-Metallochaperones

To exert its transcriptional activity, the p53 protein requires critical amounts of zinc for proper folding and binding to DNA response elements [92]. Failures in zinc binding lead to p53 destabilization and DNA binding deficiencies. p53-R175H is a prime example for a zinc-binding mutant, which inactivates p53 by reducing its affinity for zinc ions. Zinc metallochaperones (ZMC) target p53 zinc-binding mutants that have lost the ability to properly bind zinc in a new strategy. Using the NCI drug library, Yu and coworkers identified the thiosemicarbazone ZMC1, also known as NSC319726 that inhibited cancer cell growth, specifically in p53-R175 mutant tumors [93]. Mechanistically, ZMC1 restores the wild-type structure of p53-R175, its transcriptional activity, and growth inhibition. No growth inhibition was seen in tumor cells expressing wtp53 or p53-R273H. Importantly, ZMC1 also kills p53-R172H knock-in mice with extensive apoptosis and inhibits tumor growth in p53-R175H-haboring xenografts. More recently, Yu et al. presented that ZMC1 is able to reactivate other p53 mutants with impaired zinc interactions such as p53-C238S, p53-C242F and p53-C176F mutations [75]. ZMC1 does not bind its mutp53 target proteins directly. Instead, it regulates the transport of free zinc ions. ZMC1 reactivates mutp53 indirectly by increasing the available intracellular zinc, enabling these zinc-binding defective mutants to overcome their lowered affinity [94,76].

#### 2.2.6. Disrupting mutp53/TAp73 Protein Complexes

The p53 family member TAp73 shows a high degree of structural and functional homology to p53 and can form heterotetramers with mutp53. This leads to broad inhibition of p73 functions, resulting in enhanced proliferation and chemoresistance. Disruption of mutp53/p73 complexes should allow TAp73 reactivation. Indeed, RETRA, named for “reactivation of transcriptional reporter activity” [77] and prodigiosin [78] disrupt mutp53/p73 complexes and reactivate TAp73, leading to p73-mediated tumor shrinkage. The RETRA-mediated response was strongly impaired in mutp53-bearing cells when TAp73 was depleted [77]. In contrast, Prodigiosin induced TAp73 expression and disrupted its interaction with mutp53, leading to anti-tumor effects [78]. Inhibition of mutp53/p73 protein complexes to ‘release’ TAp73 suppressor function might be a promising complementary strategy to mutp53 refolding strategies in those tumor cells where such complexes are the main route for GOF activity.

Additional small molecule reactivators and stabilizers of mutp53 were described but could not be discussed here due to space limitation, for which we apologize. We refer the reader to more detailed recent reviews [6,7,19,20,95,96].

#### 2.2.7. CrispCas9-Mediated Restoration of Wild-Type p53 via Cancer Gene Therapy

The development of the CRISPR/Cas9 genome editing system in vivo, a novel TP53 therapeutic concept, could be envisioned in the future, capable of restoring the TP53wt genotype in cancer cells. One such ambitious concept idea has been proposed [97]. To cover the broad mutational distribution spanning the entire DNA-binding domain, the authors envision to replace the entire TP53mut locus (~20.5 kb in length) with a functional cDNA copy of TP53wt. Homologous recombination is directed by upstream and downstream sgRNA1 and sgRNA2 that bind to the flanking sites of the TP53mut locus. The single multiplex therapeutic vector (administered i.v.) is tumor-specific and inducible to allow spatial-temporal control and increase safety. The design is based on a delivery phage-hybrid system (AAVP vector) that is restricted to tumor cells via the survivin promoter driving Cas9 and TP53wt (survivin is upregulated in most cancer types, but undetectable in adult tissues) and functionally controlled by induction with doxycycline. In theory, this ‘universal’ TP53 reagent, covering the broad p53 mutational spectrum, should result in induction of cancer cell death and tumor regression, but awaits experimental testing.

## 3. Conclusions

p53 missense mutant alleles, present in nearly 40% of all human tumors and in 75% of all 53-altered human tumors, often act as driver oncogenes. Notably, these oncogenic gain-of-function (GOF) missense mutant p53 proteins (‘mutp53′) undergo constitutive stabilization specifically in tumors. Stabilization is a pre-requisite for their GOF. This creates exploitable tumor-specific dependencies and therapeutic vulnerabilities. The discussed strategies directly target stabilized mutp53 proteins for reactivation or degradation. Of the two classes, the pleomorphic degradation strategies are the most advanced, with impressive survival effects shown for some of them in knockin mouse models. This is in part owed to the fact that the drugs used for HSP90/HDAC6 axis and the statins are either already FDA-approved or in clinical development, while the ‘reactivators’ are mostly at the level of in vitro cell-based testing. As these concepts move into clinical trials, the diagnostics on p53 status for patient selection has to go beyond tumor sequencing and also validate mutp53 stabilization in tumor tissue by immunohistochemistry. Diagnostic-level next generation monoclonal antibodies against specific mutp53 hotspot mutations might be useful here. A first step was done by Hwang and coworkers who generated mutp53-specific antibodies against three of the most common p53 hotspot mutations [98].

## Figures and Tables

**Table 1:** Compounds targeting mutant p53.

Compound Described	Mechanism of Action	Targeting	References
PRIMA-1^Met^ (APR-246)	Converted to MQ; MQ binds to thiol groups in the core domain of mutp53 to restore wtp53 function	Restoration	Bykov 2002 [66], 2005 [67] Lambert 2009 [68], 2010 [69]
PhiKan083 or PhiKan7088	Binding to p53-Y220C specific core cavity and restore wtp53-like folding	Restoration of p53 Y220C	Boeckler 2008 [70] Liu 2013 [71]
PEITC	Unknown; p53-R172H specific; restores wtp53-like function	Restoration of p53 R172H	Aggarwal 2016 [72]
ReACp53	Small peptide; blocks amyloid-like aggregation of mutp53 to shift to wtp53-like folding state	Deaggregation, Restoration	Soragni 2016 [73]
pCAP	Small peptide; binds preferentially to mutp53 when it transiently exhibits wtp53-like conformation to shift the equilibrium towards the wtp53	Stabilize wtp53 structure	Tal 2016 [74]
ZMC-1	Metallochaperone; increases intracellular zinc level to restore zinc-deficient p53 mutants such as p53-R175H which allows proper wtp53-like folding	Restoration and activation	Yu 2012 [75] Blanden 2015 [76]
RETRA	Not well known; disrupts mutp53/p73 complexes	Activation of p73	Kravchenko 2008 [77]
Prodigiosin	Not well known; induces TAp73 expression and disrupts its interaction with mutp53	Activation of p73	Hong 2014 [78]
Chetomin (CTM)	Binds to Hsp40 and increases the binding of Hsp40 to p53 R175H which leads to restoration of wtp53 conformation	Restoration of p53 R175H	Hiraki 2015 [79]
Hsp90 inhibitors	e.g., 17AAG and Ganetespib; disrupt chaperone complexes to release and activate MDM2 and/or CHIP which degrade mutp53	Mutp53 degradation	Esser 2005 [35] Li 2011 [37] Alexandrova 2015 [15]
Statin	e.g., Lovastatin inhibits Hsp40 (by decreasing mevalonate-5-phosphate) or Cerivastatin (more potent) inhibits Hsp90 (by inhibiting HDAC6) to release and reactivate CHIP and MDM2 leading to mutp53 degradation	Mutp53 degradation	Parrales 2016 [60] Ingallina 2018 [59]
